# ErbB2, EphrinB1, Src Kinase and PTPN13 Signaling Complex Regulates MAP Kinase Signaling in Human Cancers

**DOI:** 10.1371/journal.pone.0030447

**Published:** 2012-01-18

**Authors:** Paola D. Vermeer, Megan Bell, Kimberly Lee, Daniel W. Vermeer, Byrant G. Wieking, Erhan Bilal, Gyan Bhanot, Ronny I. Drapkin, Shridar Ganesan, Aloysius J. Klingelhutz, Wiljan J. Hendriks, John H. Lee

**Affiliations:** 1 Cancer Biology Research Center, Sanford Research/University of South Dakota, Sioux Falls, South Dakota, United States of America; 2 Thomas J. Watson Research Center, IBM Research, Yorktown Heights, New York, United States of America; 3 Rutgers, The State University of New Jersey, Piscataway, New Jersey, United States of America; 4 Dana-Farber Cancer Institute, Harvard Medical School, Boston, Massachusetts, United States of America; 5 Cancer Institute of New Jersey, New Brunswick, New Jersey, United States of America; 6 Department of Microbiology, The University of Iowa, Iowa City, Iowa, United States of America; 7 Cell Biology Laboratory at the NCMLS, Raboud University Nijmegen Medical Centre, Nijmegen, The Netherlands; 8 Department of Otolaryngology/Head and Neck Surgery, Sanford Health, Sioux Falls, South Dakota, United States of America; Ottawa Hospital Research Institute, Canada

## Abstract

In non-cancerous cells, phosphorylated proteins exist transiently, becoming de-phosphorylated by specific phosphatases that terminate propagation of signaling pathways. In cancers, compromised phosphatase activity and/or expression occur and contribute to tumor phenotype. The non-receptor phosphatase, PTPN13, has recently been dubbed a putative tumor suppressor. It decreased expression in breast cancer correlates with decreased overall survival. Here we show that PTPN13 regulates a new signaling complex in breast cancer consisting of ErbB2, Src, and EphrinB1. To our knowledge, this signaling complex has not been previously described. Co-immunoprecipitation and localization studies demonstrate that EphrinB1, a PTPN13 substrate, interacts with ErbB2. In addition, the oncogenic V660E ErbB2 mutation enhances this interaction, while Src kinase mediates EphrinB1 phosphorylation and subsequent MAP Kinase signaling. Decreased PTPN13 function further enhances signaling. The association of oncogene kinases (ErbB2, Src), a signaling transmembrane ligand (EphrinB1) and a phosphatase tumor suppressor (PTPN13) suggest that EphrinB1 may be a relevant therapeutic target in breast cancers harboring ErbB2-activating mutations and decreased PTPN13 expression.

## Introduction

ErbB2 (Her2) amplification/over-expression occurs in 20-30% of breast cancers resulting in aggressive tumor behavior and poor prognosis [1], [2]. ErbB2 activation via hetero-dimerization with other family members or homo-dimerization with itself (when expressed at high levels) initiates intracellular signals that culminate in transcription of many genes regulating proliferation, survival, differentiation, invasion and metastasis. In this way, ErbB2 plays a key role in orchestrating an aggressive breast cancer phenotype [3]. The advancement of targeted therapies such as trastuzumab, a humanized anti-ErbB2 antibody, improves survival of Her2 breast cancer patients [4], [5]. However, most patients do not respond to trastuzumab (62-74%), harboring *de novo* resistance; those that do respond benefit greatly with an increased probability of surviving over five years or remaining tumor free. Regrettably, 25% of these initially responding patients later fail therapy [6]–[9]. Like *de novo* resistance, the pathways leading to acquired trastuzumab resistance remain unclear [10], [11]. While significant progress has been made in understanding the role of ErbB2 in breast cancer initiation and progression, the overwhelming resistance to trastuzumab therapy suggests that additional signaling pathways exist that circumvent ErbB2 antibody-mediated blockade. Characterizing these pathways and, more importantly, the proteins that initiate them, will define novel targets for therapeutic intervention that may re-sensitize Her2 patients to trastuzumab and improve survival.

In addition to amplification/over-expression, polymorphisms in ErbB2 at codon 655 (within the transmembrane domain) are associated with increased development of breast cancer in some populations, suggesting that changes in the ErbB2 coding sequence may also have functional consequences associated with cancer [12]–[14]. ErbB2 coding sequences may affect associations with partner proteins and subsequently alter ErbB2-mediated intracellular signaling. Thus, like trastuzumab resistance, identifying these pathways will be critical for defining therapies that block or circumvent them, improving survival.

While dominant pro-oncogenic functions like those described for the ErbB2 kinase in breast cancer occur frequently in solid tumors, alterations in phosphatases likewise occur and function as tumor suppressors. For example, the non-receptor protein tyrosine phosphatase, PTPN13 (also known as FAP1, PTPL1, PTPLE, PTPBAS, PTP1E; PTP-BL is the mouse homolog) [15]–[17] has recently been dubbed a putative tumor suppressor. PTPN13 is a multi-module containing phosphatase. Its five PDZ protein-protein interaction domains mediate associations with many cellular proteins and, as such, suggest that PTPN13 mutations may alter a variety of different cellular functions [18], [19]. PTPN13 mutations have, in fact, been identified in colorectal [16], head and neck [20] and liver cancers [17], [21]. Importantly, decreased PTPN13 expression in breast cancer correlates with decreased overall survival [22]. Moreover, we previously found that decreased PTPN13 expression synergizes with an activated ErbB2 transmembrane mutation (mNeuNT) enhancing tumor growth and invasion *in vivo* [23]. While in humans amplification/over-expression of ErbB2 is oncogenic, in animals activating transmembrane ErbB2 mutations are required for tumor growth. Thus, our *in vivo* mouse studies necessitate the use of mNeuNT, a constitutively active transmembrane ErbB2 mutation. However, the finding that human ErbB2 polymorphisms can affect breast cancer prevalence suggests that the study of transmembrane activating ErbB2 mutations in animals (in the absence of amplification/over-expression) may prove beneficial in the context of human disease. While a study by Zhu *et al.* suggests that PTPN13 regulates ErbB2 function directly by de-phosphorylating the ErbB2 signal domain [24], we have not found that in our system suggesting that PTPN13 and activated ErbB2 alone cannot account for the enhanced downstream signaling, tumor growth, and invasion evident in our published studies [23]. We therefore hypothesized that additional modifiers function in the PTPN13/ErbB2 synergy observed. We further reasoned that a PTPN13 phosphatase substrate with signaling capacities may be one such candidate molecule. Therefore, we analyzed EphrinB1 [25].

EphrinB1 belongs to a family of ligands that bind and activate Eph receptor tyrosine kinases. Ephrin ligands are unique, binding and activating signaling from their cognate receptors, and themselves becoming phosphorylated and initiating their own signaling cascades. This Ephrin specific characteristic is called “reverse” signaling. “Reverse” signaling following Eph receptor engagement constitutes the conventional signaling pathway. However, Ephrins are promiscuous in their associations and signaling occurs following non-Eph receptor interactions [26], [27]. This type of Ephrin signaling is non-conventional.

Given that EphrinB1 is a phosphatase substrate of PTPN13, decreased PTPN13 expression or functional PTPN13 mutations (both of which occur in solid tumors) likely result in increased EphrinB1 phosphorylation and subsequent signaling. In the context of breast cancer where decreased PTPN13 expression correlates with poor survival, defining the pathways activated in the absence of PTPN13 may identify critical targets for therapeutic intervention and improve survival. Thus, we hypothesized that the synergy between decrease PTPN13 and increased ErbB2 activation that drives tumor growth and invasion is mediated via EphrinB1 and, further, that EphrinB1-mediated signaling is enhanced in breast cancers with compromised PTPN13 expression. Here, we describe a novel association between ErbB2 and EphrinB1. Expression of an activated ErbB2 mutant or over-expression of wildtype ErbB2 (as in Her2 breast cancers), together with decreased PTPN13 expression or function, not only enhances complex formation but also leads to EphrinB1 phosphorylation and associated downstream signaling. In this report, we characterize this complex, the signals mediated from it, and its relevance to breast cancer. In addition, we demonstrate that this complex exists in other epithelial cells and suggest that signaling from the complex plays a functional role in other solid tumors as well.

## Results

### PTPN13 Loss occurs in epithelial cancers

**Figure 1:**
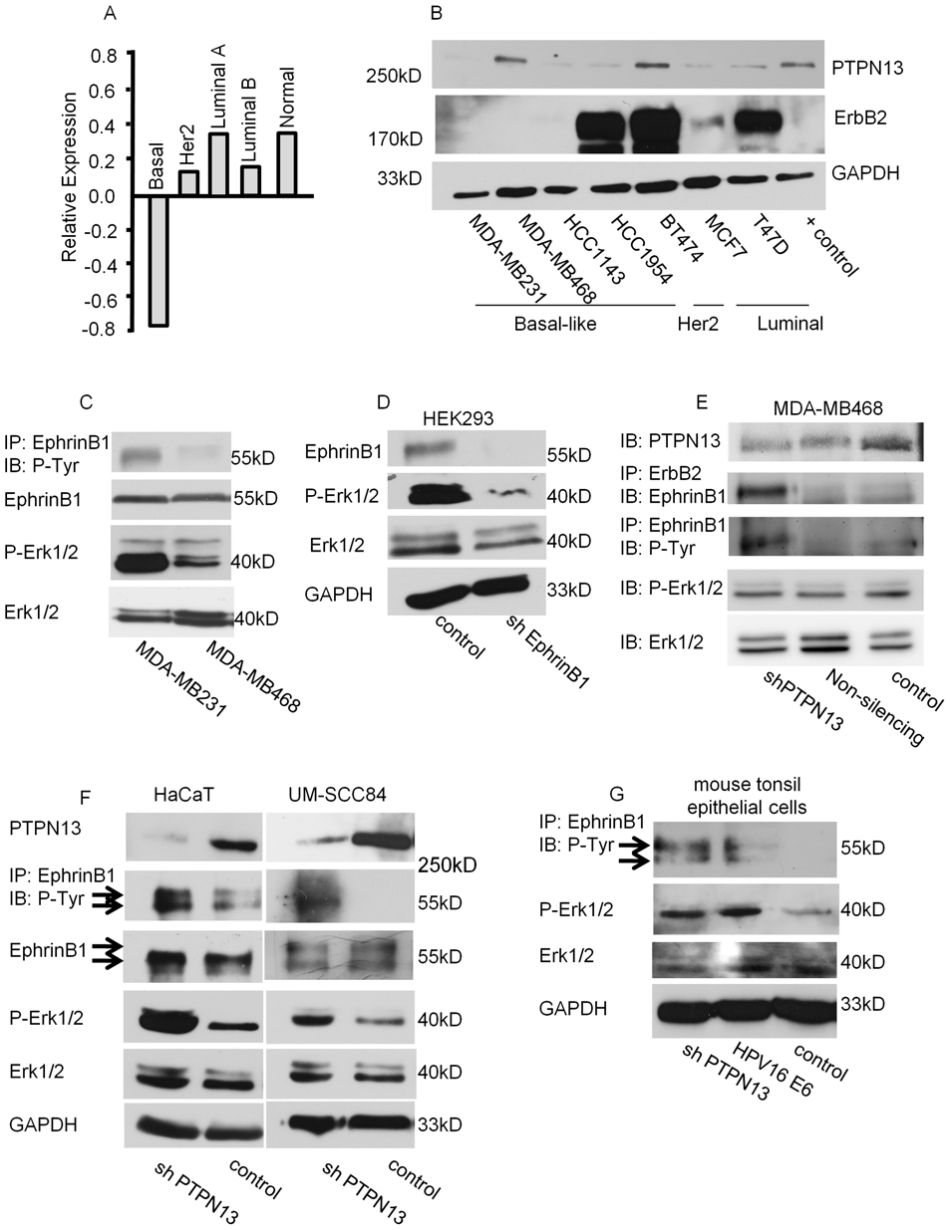
Decreased PTPN13 expression occurs in BL tumors and correlates with increased EphrinB1 and Erk1/2 signaling. (**A**) Relative PTPN13 mRNA expression of PTPN13 in molecularly characterized breast tumors. Basal-like (BL) breast cancer PTPN13 expression is decreased relative to normal breast (p = 0.00044 for basal vs. normal). (**B**) Western blot analysis of breast cancer cell lines. MDA-MB231, MDA-MB468, HCC1143, HCC1954 are breast cancer cell lines with BL breast cancer characteristics. The BT474 cell line has Her2/ErbB2 over-expressing breast cancer characteristics. MCF7 and T47D are breast cancer cell lines with luminal characteristics. HEK293 cells over-expressing PTPN13 served as a positive control. (**C**) BL breast cancer cell lines, MDA-MB231 and MDA-MB468, expressing low or high PTPN13, respectively, were analyzed by western blot. (**D**) HEK293 cells stably knocked-down for EphrinB1 (sh EphrinB1) or control were analyzed by western blot. (**E**) MDA-MB468 cells were transiently transfected with an shRNA plasmid targeting PTPN13 (shPTPN13) or a non-silencing shRNA construct (Non-silencing) and analyzed by western blot for the indicated proteins. (**F**) HaCaT cells, a human keratinocyte cell line, and UM-SCC84 cells, an HPV-negative head and neck squamous cell carcinoma cell line, stably knocked-down for PTPN13 (sh PTPN13) or control lines were analyzed by western blot. (**G**) HaCaT cells stably knocked-down for PTPN13 (sh PTPN13) or over-expressing HPV16 E6 protein (PHV16 E6) or control were analyzed by western blot for phosphorylated EphrinB1, phosphorylated Erk1/2, total Erk1/2, and GAPDH.

Decreased PTPN13 expression correlates with decreased overall survival in breast cancer [22]. We wondered whether this correlation existed across all types of breast cancers or if it was specific to a particular subtype. Thus, we analyzed a gene expression array from 200 early stage breast cancers and 7 normal breast samples for PTPN13 with particular attention to subtype specificity. While Her2, Luminal A, Luminal B breast cancers and normal breast samples express relatively high levels of PTPN13, basal-like (BL) tumors express significantly lower levels of PTPN13 mRNA (Figure 1A, p = 0.00044 for basal vs. normal). Comparisons of the other subtypes with normal breast were not significant. However, while PTPN13 mRNA levels in Her2, Luminal A and Luminal B breast cancers is not different than normal breast, the data do not eliminate the possibility that PTPN13 functional mutations occur in these subtypes that may result in a phenotype similar to that found in its absence.

We also examined PTPN13 protein expression in sub-type defined breast cancer cell lines [28]. While PTPN13 expression varied among cell lines, three out of four of the BL cell lines tested exhibited nearly absent PTPN13 protein (Figure 1B). The BL tumors comprise a heterogeneous group of cancers, but in general, are aggressive tumors with a poor prognosis [29]. Thus, our findings are consistent with those of Revillion *et al* correlating decreased PTPN13 expression and poor overall survival [22]. These data support the hypothesis that loss of PTPN13 expression impacts tumor phenotype and suggest that PTPN13 plays a role in regulating epithelial proliferation, migration and/or invasion in BL breast cancer.

### PTPN13 loss increases phosphorylated EphrinB1 and Erk1/2

PTPN13′s five PDZ domains mediate associations with many proteins, including EphrinB1 [19]. Following binding, PTPN13 de-phosphorylates EphrinB1, shutting off reverse signaling [18], [19]. To test the effects of decreased/lost PTPN13 on EphrinB1 phosphorylation, we examined two BL breast cancer cell lines: MDA-MB231, expressing nearly undetectable PTPN13 protein, and MDA-MB468, expressing endogenous PTPN13 protein (Figure 1B). As expected, low PTPN13 expression (MDA-MB231) correlates with increased EphrinB1phosphorylation while endogenous PTPN13 expression (MDA-MB468) correlates with low phospho-EphrinB1 (Figure 1C). These data are consistent with EphrinB1 being a PTPN13 phosphatase substrate and suggest that decreased PTPN13 expression in BL breast cancer cell lines increases phosphorylation of EphrinB1.

Given EphrinB1′s ability to signal, we further asked whether phosphorylated EphrinB1 correlated with increased downstream signaling. Molecular analysis of BL breast carcinomas shows that many gene products in the BL cluster are associated with MEK/Erk activation, thus we chose to analyze the phosphorylation status of Erk1/2 in these BL cell lines [30]–[33]. We found that decreased/absent PTPN13 expression (MDA-MB231) correlates with increased phosphorylation of Erk1/2 (Figure 1C); while endogenous PTPN13 expression (MDA-MB468) correlates with decreased Erk1/2 phosphorylation. These data suggest that EphrinB1 activation (phosphorylation) signals via the MAP Kinase pathway. To test this, we stably knocked-down EphrinB1 in HEK293 cells, chosen due to their ease of transfection relative to breast cancer cell lines. Knock-down of EphrinB1 results in prominent attenuation of phosphorylated Erk1/2 (Figure 1D) consistent with EphrinB1-mediated Erk1/2 activation. Taken together, the data suggest that the absence of PTPN13 results in enhanced EphrinB1 activation and concomitant Erk1/2 phosphorylation.

As a further test, endogenous PTPN13 was transiently knocked-down in MDA-MB468 cells (shRNA-mediated, shPTPN13). As predicted, PTPN13 knock-down increased phosphorylation of EphrinB1 consistent with PTPN13 regulation of EphrinB1 phosphorylation (Figure 1E) [25]. Moreover, increased EphrinB1 associated with ErbB2 in lysates from shPTPN13 cells suggesting that phosphorylated EphrinB1 associates more readily with ErbB2 as compared to unphosphorylated EphrinB1. Surprisingly, PTPN13 knock-down did not affect Erk1/2 phosphorylation, suggesting that either EphrinB1 does not signal via the MAP Kinase pathway in MDA-MB468 cells or that its signaling is modulated in these cells via additional (as yet undefined) components.

Previous attempts by our laboratory to over-express PTPN13 have been unsuccessful as its increased expression results in cell death, thus limiting our ability to analyze its downstream effects [34]. Therefore, we were unable to test the effects of over-expressing PTPN13 in MDA-MB231 cells which lack endogenous expression. However, given its affects on EphrinB1 phosphorylation in breast cancer cells, we speculated that a reduction in PTPN13 expression or function may be a common and, more importantly, a key alteration in other epithelial cancers. To test this concept, we knocked-down PTPN13 in a human keratinocyte cell line (HaCaT cells) and analyzed its affects on signaling. Decreased PTPN13 expression indeed enhanced EphrinB1 and Erk1/2 phosphorylation (Figure 1F, HaCaT). Similarly, knock-down of PTPN13 in the head and neck squamous cell carcinoma cell line, UM-SCC84, resulted in increased EphrinB1 and Erk1/2 phosphorylation (Figure 1F, UM-SCC84). Importantly, previous studies focused on human papillomavirus (HPV)-associated head and neck cancers demonstrate that the HPV16 E6 oncoprotein binds and targets PTPN13 for degradation [34], [35]. Thus, HPV positive cells served as an additional test of the function of PTPN13 in cellular signaling in the context of *virally*-mediated cancer. Thus, we analyzed previously characterized mouse tonsil epithelial cells stably expression HPV16 E6 or those stably knocked-down for PTPN13 [34], [35]. Indeed, HPV16 E6 expression enhanced EphrinB1 and Erk1/2 phosphorylation, consistent with decreased/lost PTPN13 expression. Moreover, knock-down of PTPN13 in mouse tonsil epithelial cells demonstrated a similar effect (shPTPN13, Figure 1G). Taken together, these data suggest that decreased PTPN13 expression enhances EphrinB1 and Erk1/2 phosphorylation in epithelial cells. The finding that high risk HPV viruses have evolved a mechanism to eliminate cellular PTPN13, further emphasizes the importance of PTPN13 regulatory functions critical in cellular signaling pathways. The data suggest that PTPN13 expression may be interesting to evaluate in many, if not all, solid tumors.

### ErbB2 co-immunoprecipitates and co-localizes with EphrinB1

**Figure 2:**
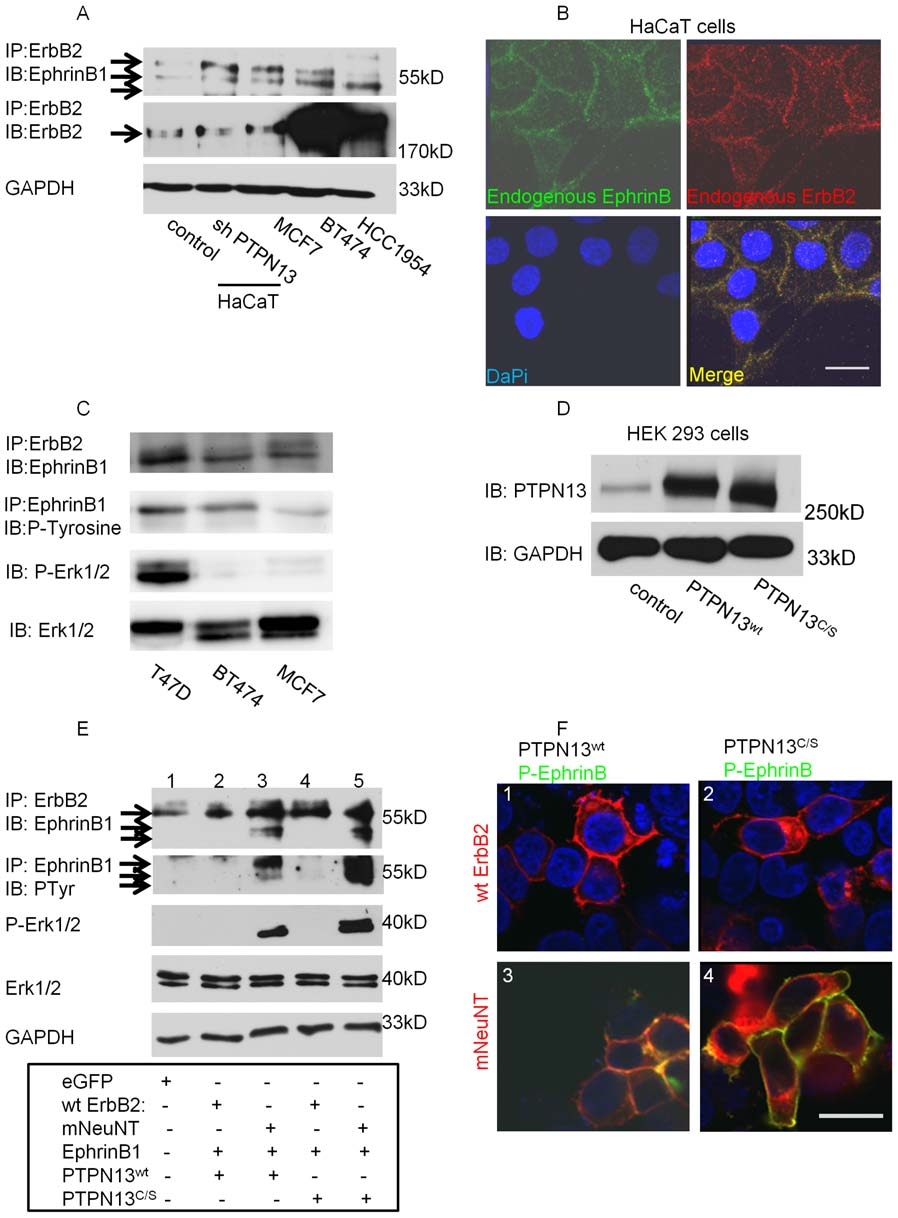
mNeuNT is required for EphrinB1 activation and initiation of signaling. (**A**) Western blot analysis of a human keratinocyte cell line, HaCaT cells (control as well as cells knocked-down for PTPN13), and breast cancer cell lines (MCF7, BT474, HCC1953) immunoprecipitated (IP) for ErbB2 and immunoblotted (IB) for EphrinB1. Membrane was re-probed for ErbB2. GAPDH was used as a loading control. (**B**) *En face* confocal images of HaCaT cells immunolocalizing surface EphrinB (green) and total ErbB2 (red). Nuclei are counterstained with DaPi (blue). Scale bar 20 µm. (**C**) Western blot analysis of breast cancer cell lines: T47D, BT474 and MCF7. (**D**) HEK293 cells transiently transfected with either wildtype PTPN13 or the C/S PTPN13 mutant analyzed by western blot. (**E**) HEK293 cells transiently transfected with either eGFP alone, or a combination of EphrinB1, ErbB2 (wildtype or mNeuNT), and PTPN13 (wildtype or C/S mutant) and analyzed by western blot. (**F**) *En face* confocal images of cells transfected in **E** processed for immunolocalization of phosphorylated EphrinB (green) and ErbB2 (red). Nuclei counterstained with DaPi (blue). Scale bar 20 µm.

The above data suggest that decreased/lost PTPN13 increases EphrinB1 activation which may then modulate downstream phosphorylation of Erk1/2. Our laboratory has previously demonstrated that decreased/lost PTPN13 synergizes with ErbB2, potentiating MAP Kinase signaling [23]. Thus, we wondered whether EphrinB1 phosphorylation and the resulting Erk1/2 signaling occurs in an ErbB2-mediated, non-conventional manner. Therefore, we asked whether EphrinB1 associates with ErbB2 and completed co-precipitation and co-localization studies. We found that EphrinB1 and ErbB2 co-precipitate (Figure 2A) from lysates derived from breast cancer cell lines as well as HaCaT cells. Interestingly, knock-down of PTPN13 in HaCaT cells (shPTPN13) enhanced pull-down of EphrinB1 with ErbB2, again suggesting that phosphorylated EphrinB1 associates more readily with ErbB2 than the unphosphorylated state. In addition, in all cases multiple forms of EphrinB1 were pulled down with ErbB2 (Figure 2A arrows). We speculate these bands represent different phosphorylated forms, as suggested by Xu *et al*. In their study, mutation of EphrinB1 tyrosine residues results in the specific loss of EphrinB1 bands suggesting that the bands evident by western blot represent phosphorylated forms of the protein [36]. Alternatively, the bands may represent unglycosylated or degraded EphrinB1 as suggested by Makarov *et al* [37]. While the identity of these bands is undefined in this study, different EphrinB1 antibodies verified its co-immunoprecipitation with ErbB2 (data not shown). Importantly, while varying amounts of ErbB2 were pulled down in all lysates tested (consistent with their different levels of ErbB2 expression), a similar amount of EphrinB1 was associated with it. These data suggest that there is a limit to the amount of EphrinB1 that associates with ErbB2; more ErbB2 expression does not result in increased EphrinB1 association. These data suggest that the interaction is tightly regulated.

Co-immunostaining of endogenous ErbB2 and endogenous, surface EphrinB in HaCaT cells shows that ErbB2 and EphrinB co-localize at cell-cell junctions (Figure 2B). Surface EphrinB was localized on unfixed, unpermeabilized cells using EphB1-Fc. EphB1-Fc is a chimera consisting of the extracellular region of the EphB1 receptor (a cognate EphrinB receptor) fused to human IgG_1_. Thus, EphB1-Fc binds to surface expressed EphrinB ligands. These interactions are then detected using an anti-human IgG-FITC and cells analyzed by confocal microscopy. Thus, while the surface staining is not specific to EphrinB1alone, it does suggest that EphrinB proteins co-localize with ErbB2. Together with the immunoprecipitation data, these data suggest that EphrinB1 associates with ErbB2.

To assess the significance of the ErbB2/EphrinB1 interaction in breast cancer, we further analyzed: BT474, a Her2 (ErbB2) cell line; T47D, a luminal cell line with high ErbB2 expression; MCF7, another luminal cell line with low ErbB2 expression (Figure 1B). Interestingly, both T47D and MCF7 cells express nearly undetectable PTPN13, while BT474 cells express endogenous PTPN13 (Figure 1B). Immunoprecipitation for ErbB2 followed by western blot analysis for EphrinB1 demonstrates enhanced EphrinB1 co-IP in T47D cells which correlated with higher levels of phosphorylated EphrinB1. This finding is consistent with our previous data suggesting that phosphorylated EphrinB1 associates more readily with ErbB2. In addition, T47D cells demonstrate robust phosphorylation of Erk1/2 which was undetectable in BT474 and MCF7 cells (Figure 2C). Taken together, these data suggest that in breast cancer cell lines with low/absent PTPN13 expression, and high ErbB2 expression, EphrinB1 phosphorylation is elevated as is its association with ErbB2 and correlates with enhanced Erk1/2 phosphorylation. The data further suggest that lack of effect on Erk1/2 phosphorylation in shPTPN13 MDA-MB468 cells (Figure 1E) may be due to insufficient ErbB2 expression and/or complex formation with EphrinB1.

### mNeuNT increases complex formation and signaling

Given that ErbB2 is a tyrosine kinase and EphrinB1 phosphorylation initiates reverse signaling, we wondered whether ErbB2 phosphorylates EphrinB1. In addition, given its affects on EphrinB1 phosphorylation, we speculated that PTPN13 regulates this activation. To examine this, both wildtype PTPN13 (PTPN13^wt^) as well as a phosphatase null mutant (PTPN13^C/S^) were tested. In addition, our previous findings demonstrate that a constitutively active ErbB2 transmembrane mutant (V660E, henceforth referred to as mNeuNT) synergizes with loss of PTPN13 and increases MAP Kinase signaling and invasive growth whereas wildtype (endogenous) ErbB2 does not [23]. Therefore, both mNeuNT and wildtype ErbB2 (wt ErbB2) were also tested. Given their low endogenous expression of PTPN13, ease of transfection and robust expression of transfected PTPN13, HEK293 cells were utilized for these studies (Figure 2D). In these experiments HEK293 cells were transiently transfected with ErbB2 (either wildtype or mNeuNT), wildtype EphrinB1 and PTPN13 (either wildtype or the C/S mutant) and analyzed by western blot.

Control lysates (eGFP) show that EphrinB1 co-immunoprecipitates with ErbB2 but that EphrinB1 is not phosphorylated and Erk1/2 is not activated (lane 1, Figure 2E). Expression of neither wildtype (lane 2, Figure 2E) nor C/S PTPN13 (lane 4, Figure 2E) changes these parameters in the presence of over-expressed wt ErbB2 and EphrinB1. In contrast, expression of mNeuNT with EphrinB1 increases not only the amount of EphrinB1 associating with it, but also leads to EphrinB1 and Erk1/2 phosphorylation (lanes 3 and 5, Figure 2E). HEK293 cells express little endogenous ErbB2 (data not shown); in addition, the anti-ErbB2 antibody utilized for immune precipitation recognizes both wildtype ErbB2 and mNeuNT. Thus, while co-IP studies cannot distinguish between EphrinB1 associated with endogenous ErbB2 or mNeuNT, the data strongly support an association mNeuNT. While we have previously demonstrated that mNeuNT expression alone increases Erk1/2 phosphorylation [23], our finding that expression of PTPN13^C/S^ is not capable of reducing EphrinB1 and Erk1/2 phosphoryaltion , suggests that EphrinB1-mediated reverse signaling also contributes to Erk1/2 phosphorylation (Figure 2E, lane 5, arrows). In addition, expression of wildtype PTPN13 (lane 3, Figure 2E), but not C/S PTPN13 mutant (lane 5, Figure 2F), decreases the amount of phosphorylated EphrinB1 and P-Erk -1/2, also consistent with EphrinB1 phosphorylation affecting MAP Kinase signaling.

These biochemical data were confirmed by immunolocalization studies of phosphorylated EphrinB (Figure 2F, green) and ErbB2 (Figure 2F, red). Only expression of mNeuNT results in phosphorylated EphrinB present at the cell surface (Figure 2F, panels 3 and 4, yellow indicates expression co-localization of phosphorylated EphrinB and ErbB2). Moreover, only wildtype PTPN13 decreases the amount of phosphorylated EphrinB at the cell surface (Figure 2F, compare yellow and green between panels 3 and 4). Taken together, these data suggest that, 1) mNeuNT associates with EphrinB1 and this association is enhanced with EphrinB1 phosphorylation, 2) phosphorylated EphrinB1 correlates with phosphorylation of Erk1/2 and 3) that PTPN13 de-phosphorylates EphrinB1 in this context.

### mNeuNT co-immunoprecipitates with and activates Src

**Figure 3:**
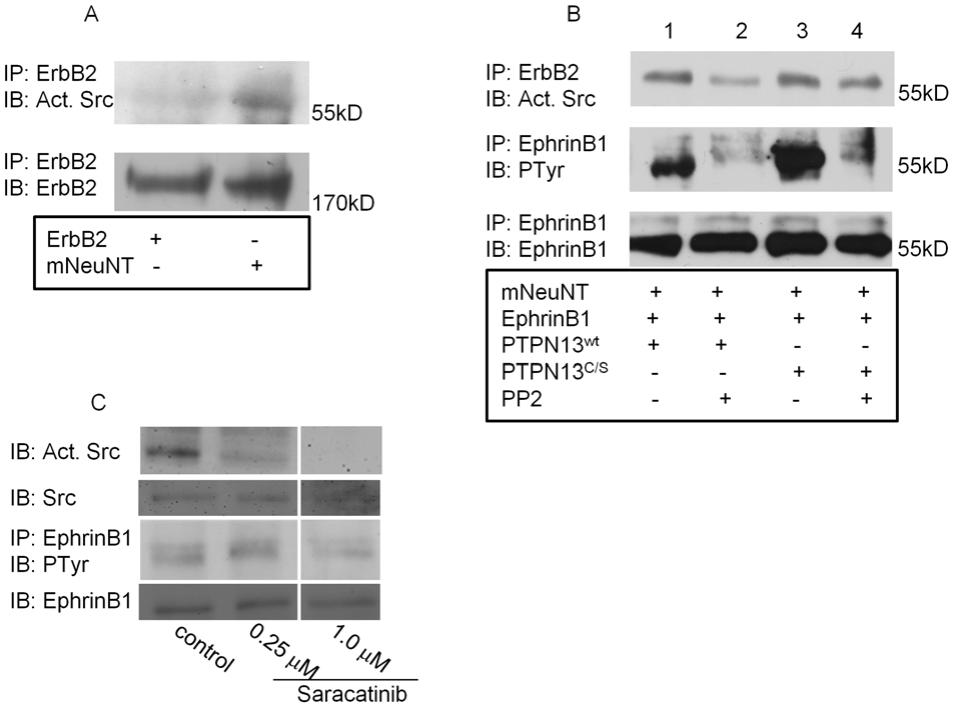
mNeuNT associates with activated Src which phosphorylates EphrinB1. (**A**) HEK293 cells transiently transfected with either wildtype ErbB2 or mNeuNT and analyzed by western blot. (**B**) HEK293 cells transiently transfected with a combination of EphrinB1, mNeuNT, and PTPN13 (wildtype or C/S mutant) were treated with or without PP2 and analyzed by western blot. (**C**) Untransfected HEK293 cells treated with increasing doses of saracatinib and analyzed by western blot for expression of endogenous activated Src, total Src, phosphorylated EphrinB1 and immunoprecipitated EphrinB1.

ErbB2-mediated signaling occurs directly via its kinase activity or by its recruitment of Src into a signaling complex [38]. Moreover, following binding to its cognate Eph receptor, Src phosphorylates EphrinB1 [25]. Since both wildtype ErbB2 and mNeuNT contain a wildtype tyrosine kinase domain, we hypothesized that the enhanced EphrinB1 phosphorylation and MAP Kinase signaling evident in the context of decreased/lost PTPN13, involves Src. Thus, we first set out to determine whether Src associates with ErbB2, as suggested by the literature [38]. HEK293 cells were transiently transfected with wildtype ErbB2 or mNeuNT and tested. While co-IP of activated Src with wildtype ErbB2 was nearly undetectable, activated Src associated with mNeuNT (Figure 3A). The anti-activated Src antibody recognizes Src tyrosine 416 (pSrc-Y416) when phosphorylated, a site that promotes Src activity [39], [40]. These data suggest that mNeuNT associates with activated Src.

### Src mediates EphrinB1 phosphorylation

Both mNeuNT and Src are kinases, either of which may phosphorylate EphrinB1. In addition, mNeuNT preferentially associates with activated Src (Figure 3A). Therefore, we tested whether activated Src (rather than mNeuNT) mediates EphrinB1 phosphorylation. HEK293 cells were transiently transfected with mNeuNT, EphrinB1 and either wildtype or mutant (C/S) PTPN13 and analyzed by western blot. Consistent with the above data, mNeuNT co-IPs with activated Src and EphrinB1 is phosphorylated (Figure 3B, lane 1); PTPN13^C/S^ enhances EphrinB1 phosphorylation (Figure 3B, lane 3). To test the role of Src in EphrinB1 phosphorylation, transfected cells were treated with PP2, a potent Src inhibitor. Xu *et al* previously demonstrated that treatment with 1 µM PP2 efficiently blocks Src-mediated EphrinB1 phosphorylation while treatment with 25 µM PP2 results in cell detachment [36]. Thus, in this study to ensure efficient Src inhibition, cells were treated with 10 µM PP2 for a short time (4 hours). In mNeuNT, EphrinB1 and wildtype PTPN13 transfected lysates, PP2 treatment decreased the amount of activated Src associated with mNeuNT and attenuates EphrinB1 phosphorylation (Figure 3B, lane 2). Lysates of mNeuNT, EphrinB1 and PTPN13^C/S^ transfected cells were similarly affected by PP2 suggesting that EphrinB1 phosphorylation within the mNeuNT, Src, PTPN13 complex is mediated via Src. Taken together, these data suggest that Src, rather than mNeuNT, phosphorylates EphrinB1 and further supports the published literature and our own findings that PTPN13 is responsible for de-phosphorylating EphrinB1 in this complex.

PP2 is a Src-family kinase inhibitor, blocking activation of Lck, Fyn, Hck and Src. In addition, the experiments performed using PP2 utilized HEK293 cells over-expressing PTPN13, ErbB2 and EphrinB1. Thus, to more selectively inhibit Src and to test its function on phosphorylation of endogenous EphrinB1, we also analyzed saracatinib (AZD-0530, currently in clinical trials [41]–[44]) on non-transfected cells. HEK293 cells were treated with saracatinib (0, 0.25 µM or 1.0 µM) and analyzed by western blot. Saracatinib treatment successfully inhibited Src activation and a dose response was evident. In addition, at the highest dose, there was a decrease in the amount of EphrinB1 phosphorylation (Figure 3C) consistent with a role for Src in mediating EphrinB1 phosphorylation.

### EphrinB1 immunoprecipitates with ErbB2 in a manner that does not require the extracellular or C-terminal PDZ motif

Our data suggest that regulation of the ErbB2/EphrinB1 complex may mediate signals important in breast cancer. Given that ErbB2 and EphrinB1 interact, rationale design of small molecule inhibitors to block their association may be of therapeutic value. Thus, ErbB2 and EphrinB1 mutants were generated to define the domains necessary and sufficient for their association.

**Figure 4:**
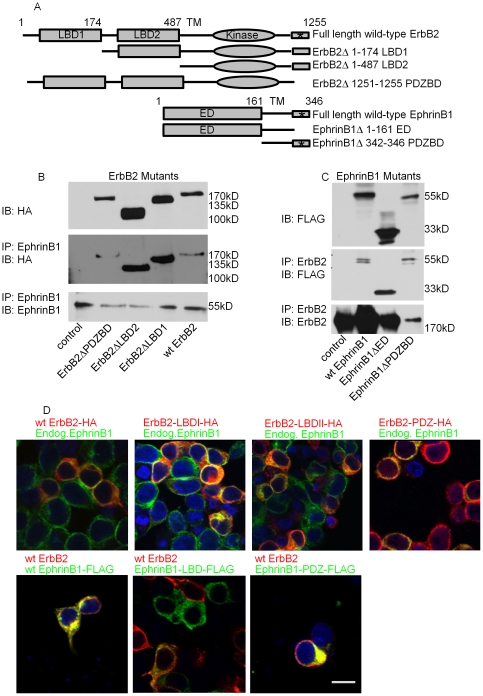
ErbB2 and EphrinB1 associate in a manner that likely requires the transmembrane domains. (**A**) Schematic representation of ErbB2 and EphrinB1 mutants. LBD, ligand binding domain. * , PDZ binding domain (PDZBD). Numbers refers to amino acid number. (**B**) HEK293 cells were transiently transfected with HA-tagged wildtype or mutant ErbB2 constructs and analyzed by western blot. (**C**) HEK293 cells were transiently transfected with FLAG-tagged wildtype or mutant EphrinB1 constructs and analyzed by western blot. (**D**) HEK293 cells transfected in **B** and **C** were further analyzed by immunofluorescence and confocal microscopy. *En face* confocal images of immuno-localized HA-tagged ErbB2 (red, top panels) and endogenous EphrinB1 (endog. EphrinB1,green, top panels). Yellow color signifies co-localization. Immunostaining of FLAG-tagged EphrinB1 (green, bottom panels) and wild-type ErbB2 (red, bottom panels). Yellow color signifies co-localization. Nuclei were counterstained with DaPi (blue). Scale bar 20 µm.

ErbB2 contains two large extracellular domains which we designated ligand binding domains 1 and 2. ErbB2 extracellular mutants deleted of either ligand binding domain 1 (Δ 1–174 LBD1) or both domains 1 and 2 (Δ 1–487 LBD2) were generated. ErbB2′s PDZ binding domain was deleted in a third mutant (Δ 1251–1255 PDZBD) (Figure 4A). All ErbB2 mutants, including the full length wildtype protein, were HA tagged at the N-terminus. Constructs were transfected into HEK293 cells and analyzed for loss of co-IP with endogenous EphrinB1. All constructs express HA-tagged proteins that run at predicted molecular weights. Endogenous EphrinB1 associated with all ErbB2 mutants suggesting that none of the deleted domains were essential for the interaction (Figure 4B).

Wildtype and mutant EphrinB1 constructs were generated and FLAG tagged at the N-terminus. EphrinB1 was deleted either of its entire extracellular domain (Δ 1–161 ED) or only its PDZ binding domain (Δ342–346 PDZBD, Figure 4A). HEK293 cells were transfected and analyzed. All constructs express FLAG-tagged proteins that run at the predicted molecular weights. Again, no loss of co-IP between wildtype ErbB2 and the EphrinB1 mutants occurred (Figure 4C). In addition, transfected cells were studied by immunofluorescence and confocal microscopy. All HA-tagged ErbB2 constructs localized to the membrane with endogenous EphrinB1. Similarly, all FLAG-tagged EphrinB1 constructs retained co-localization with wildtype ErbB2 (Figure 4D). These data suggest that the ErbB2/EphrinB1 association is not mediated via the extracellular or PDZ binding domains of either partner but rather that their transmembrane domains, ErbB2′s kinase domain or remaining intervening sequences (retained in all mutants), alone or together, play a key role in the interaction. Future studies mutating these domains will define the critical elements mediating the ErbB2/EphrinB1 association.

## Discussion

We describe a complex consisting of ErbB2, Src, EphrinB1 and PTPN13 that mediates EphrinB1 phosphorylation and downstream signaling in breast cancer cells. In addition, we present similar findings using multiple human cell lines suggesting that complex formation and signaling occurs in many, if not all, epithelial cells. With respect to breast cancer, ErbB2/EphrinB1 signaling may be most relevant in tumors with high ErbB2 expression and either low/absent PTPN13 expression or those harboring PTPN13 functional mutations. Our study and those of others predict that these tumors possess an aggressive phenotype and poor prognosis [22], [45].

In the breast cancer cell lines studied, low/absent PTPN13 together with elevated ErbB2 expression correlate with enhanced ErbB2/EphrinB1 association as well as increased EphrinB1 and Erk1/2 phosphorylation. Interestingly, both MDA-MB231 and MDA-MB468 cells lack detectable (by western blot) ErbB2 expression yet, in the absence of PTPN13, EphrinB1 is phosphorylated. Both of these BL breast cancer cell lines demonstrate over-expression of ErbB1 [30], [46] suggesting that ErbB1 may hetero-dimerize with low levels of endogenous ErbB2 (forming an ErbB1/ErbB2/EphrinB1 complex) and mediate signaling from the complex. It remains unclear why transient knock-down of PTPN13 in MDA-MB468 cells failed to increased Erk1/2 phosphorylation (Figure 1E) though a few possibilities exist. First, while the extent of PTPN13 knock-down was not very efficient, it was enough to increase EphrinB1 phosphorylation (Figure 1E). This suggests that either EphrinB1 does not signal via the MAP Kinase pathway, or that the specific EphrinB1 tyrosine(s) necessary to mediate such signaling were not activated under this condition. Our data demonstrating that knock-down of EphrinB1 greatly attenuates Erk1/2 phosphorylation (Figure 1D) argue against the former possibility and support the latter. Second, the presence of high ErbB1 expression in MDA-MB468 cells and the fact that these cells were not serum-starved suggests that ErbB1 signaling (either alone or in combination with low level endogenous ErbB2) modulates downstream pathways which include Erk1/2. Third, it is possible that the ErbB2/EphrinB1 complex is composed of additional components (in fact, we hypothesize this is true), the composition of which may differ among different cell lines and may respond differently under different contexts. Further characterization of the ErbB2/EphrinB1 complex, its association with additional transmembrane proteins (including ErbB family members), as well as intracellular binding partners and the signaling pathways they regulate are on-going and will increase our understanding of the function of this complex in breast cancer. What is clear from these experiments is that EphrinB1 associates with ErbB2 and its phosphorylation is regulated by PTPN13. The absence of PTPN13, as occurs in BL breast cancers, affects EphrinB1 phosphorylation and likely downstream signaling, which may include components of the MAP Kinase pathway.

Given its over-expression in Her2 breast cancers, we further studied the ErbB2/EphrinB1 association in additional breast cancer cell lines. T47D cells which express high levels of ErbB2 and nearly undetectable PTPN13, demonstrate increased ErbB2/EphrinB1 association, elevated EphrinB1 activation and robust Erk1/2 phosphorylation. BT474 cells which demonstrate robust ErbB2 expression and high PTPN13 expression, lack Erk1/2 phosphorylation while MCF7 cells, with low expression of both ErbB2 and PTPN13, also have undetectable phosphorylation of Erk1/2 (Figure 2C). These data suggest that the combination of low/absent PTPN13 and high ErbB2 expression are required for driving EphrinB1 and Erk1/2 phosphorylation.

Our studies also demonstrate that, whereas PTPN13 is the phosphatase silencing EphrinB1 activation, Src is the kinase that activates it (Figure 3B, 3C). Thus, signaling from the ErbB2/EphrinB1 complex is regulated by transient associations with PTPN13 and Src. The interaction of two known kinase oncogenes (ErbB2 and Src), a signaling ligand (EphrinB1) and a putative tumor suppressor phosphatase (PTPN13) likely regulates key signals not limited to Erk1/2. In cancers where protein kinase oncogene expression and/or signaling are enhanced, signals mediated from this complex may be altered. Our data in breast cancer cell lines support this hypothesis.

The finding that wildtype ErbB2 associates with EphrinB1 but does not correlate with EphrinB1 or Erk1/2 phosphorylation, suggests that in the absence of ErbB2 activation, Src either does not associate with the complex or remains in an inactive form. In fact, we show that mNeuNT (but not wildtype ErbB2) associates with activated Src, consistent with a requirement for activated ErbB2 to initiate complex signaling. These findings are highly relevant not only for Her2 breast cancers but also for epithelial cancers harboring activating ErbB2 mutations.

mNeuNT association with activated Src suggests that ErbB2 sequence alterations not only enhance complex formation but also initiate its signaling. Both mNeuNT and the human ErbB2 codon 655 polymorphism are single amino acid changes within the transmembrane domain of ErbB2 [47]–[49] suggesting that the transmembrane domain mediates critical interactions in disease. Interestingly, low grade *in situ* lesions of the cervix are not associated with this polymorphism [50] while advanced cervical cancer is strongly associated with it [51]. In head and neck squamous cell carcinoma, codon 655 polymorphism is associated with malignancy [52]. Our current findings suggest that ErbB2 transmembrane mutations (like neu (rat) and the human 655 polymorphism) synergize with decreased/lost PTPN13, allowing breast cancer progression via a mechanism involving increased ErbB2/EphrinB1 signaling. Our ErbB2/EphrinB1 truncation experiments also support a functional role for the transmembrane domains. In fact, a functional role for ErbB transmembrane domains has been previously described [53]–[55]. Our finding that a single amino acid change within ErbB2′s transmembrane domain (mNeuNT) increases its association with EphrinB1, robustly activating complex signaling, supports these published data. The association of human ErbB2 transmembrane polymorphism (codon 655) with cancer is also consistent with this role.

Interestingly, while expression of mNeuNT is required for Erk1/2 phosphorylation in transiently transfected HEK293 cells (Figure 2E), in retrovirally infected HEK293 cells, Erk1/2 phosphorylation occurs in the absence of mNeuNT (Figure 1D). These data suggest that retroviral infection and integration mediates cellular changes not evident in transient plasmid transfections. Despite this, the stable knock-down of EphrinB1 attenuates Erk1/2 phosphorylation consistent with EphrinB1 signaling via the MAP Kinase pathway.

The activated Src antibody used in our studies recognizes tyrosine 416 when phosphorylated; phosphorylation of this residue promotes Src activity [39]. Glondu-Lassis *et al* showed in the mouse that PTPN13 directly de-phosphorylates this tyrosine residue, inactivating Src [45]. We find no significant difference between the amount of activated Src associated with mNeuNT in the presence of wildtype or C/S PTPN13 suggesting that the cellular pool of Src associated with ErbB2 may be insensitive or inaccessible to PTPN13-mediated inactivation (Figure 3B, lanes 1 and 3).

The existence of this complex in breast cancer cells suggests that therapies targeting one component will likely be insufficient at blocking all cellular signaling mediated by the complex. For example, while blocking ErbB2 with trastuzumab (Herceptin) may effectively block ErbB2-mediated signals, it may not alter EphrinB1-mediated signaling from the complex. Our data demonstrate that in the absence of EphrinB1, Erk1/2 phosphorylation is greatly attenuated (Figure 1D) suggesting not only that EphrinB1 is a good therapeutic target, but that blocking EphrinB1 together with ErbB2 may efficiently inhibit complex signaling. Our data further suggest that breast cancers with elevated ErbB2 expression and compromised PTPN13 expression and/or function would benefit the most from this type of dual targeting.

Decreased PTPN13 expression in BL breast cancers supports a tumor suppressive role for PTPN13 (Figure 1A). BL breast cancers do not express the estrogen or progesterone receptors nor do they over-express ErbB2 [56]–[58]. Thus, these patients do not benefit from targeted therapies, contributing to their poor outcome. Moreover, while only Her2 breast cancers demonstrate over-expression/amplification of Her2, the other breast cancer subtypes express Her2, albeit at normal levels. However, our data suggest that it is the combination of increased ErbB2 together with compromised PTPN13 that is necessary for EphrinB1 activation and downstream signaling. Despite this, MDA-MB231 cells demonstrate EphrinB1 activation and robust Erk1/2 phosphorylation (Figure 1C). The key to these data may lie in the fact that this cell line (as well as MDA-MB468) expresses high levels of ErbB1 suggesting that EphrinB1 associates with additional ErbB family members. We have some preliminary data suggesting this is indeed the case (data not shown). Thus, complex formation in BL breast cancers is likely to occur with downstream signaling amplified in the absence of PTPN13 and over-expression of ErbB1 or other ErbB family members.

This is the first study to describe a novel complex between ErbB2 and EphrinB1 that is not restricted to breast cancer cell lines but is present in many epithelial cell lines tested. Importantly, ErbB2/EphrinB1 interactions may occur *in cis* (within the same cell) or *in trans* (across neighboring cells) mediating dual or bi-directional signals, respectively. The concept of dual/bi-directional signaling in the arena of breast cancer is new and untested with potential implications for tumor growth. Further studies focused on identifying additional components of the ErbB2/EphrinB1 complex and the downstream pathways they regulate may identify additional targets for therapeutic intervention in breast cancer and other solid tumors.

## Materials and Methods

### Short hairpin RNA (shRNA) vectors

shRNA vectors were obtained from Open Biosystems (Huntsville, AL). Retrovirus was produced as previously described [59], [60]. Alternatively, shRNA plasmids were transfected into cells (Polyfect Transfection Reagent, Qiagen, Valencia, CA) as per manufacturer's directions.

### Plasmids

mNeuNT and PTPN13^C/S^ have been previously described [23]. Full length wildtype human EphrinB1 cDNA was obtained from Open Biosystems (IHC1380), sequence verified, cloned by PCR into the p3XFLAG CMV vector (Sigma-Aldrich, St. Louis, MO) by using the KpnI/XbaI sites. EphrinB1 mutants were generated by PCR and ligated into p3XFLAG CMV using NotI/SalI sites. The wildtype and mutant ErbB2 were generated by PCR and ligated into pCMV-HA (Clontech, Mountain View, CA). All final plasmids were sequence verified. Primers were as follows: Full length wildtype ErbB2: Forward: acgagtcgacgatggagctgcgcgccttg, Reverse: aaggaaaaaagcggccgctcacactggcacgtccagacc; ErbB2 LBD1: Forward: gcgatagcggtcgactagacacaagaacaaccagctggct, Reverse: gcgatagcggcggccgctcacactggcacgtccaga; ErbB2 LBD2: Forward: gcgatagcggtcgactatgcggaacccgcaccaagct, Reverse: gcgatagcgctcgagtcacactggcacgtccagacc; ErbB2 PDZBD: Forward: gcgatagcggtcgaccatggagctggcggcctt, Reverse: gcgatagcggcggccgctcaacccaggtactctgggttctctg ; Full length wildtype EphrinB1: Forward primer: gcgatagcgggtaccgggaagatggctcggcct, Reverse primer: gcgatagcggaattctcagaccttgtagtagatgttcgccg; EphrinB1 LBD: Forward primer: gcgatagcggcggccgcatgaaggttgggcaagatcc, Reverse primer: gcgatagcgtctagatcagaccttgtagtagatgttcgc; PDZBD: Forward primer: gcgatagcggcggccgcgggaagatggctcggcct, Reverse primer: gcgatagvgtctagatcactcttggacgatgtagacaggg.

### Cell culture

MDA-MB468, MDA-MB231, MCF7, 4T1, CT26, HEK293, BT474, T47D, HCC1954 and HCC1143 were obtained from the American Tissue Culture Collection (ATCC). HaCaT cells were obtained from Cell Lines Service. The UM-SCC84 was a kind gift from Dr. Douglas Trask (University of Iowa). This human cell line was originally generated at the University of Michigan by the Head and Neck SPORE Translational Research group. This group has completed genotyping of 73 UM-SCC cell lines which has been published [61]. While the UM-SCC84 line was not included in the 73 genotyped cell lines, results from continued efforts to genotype remaining and newly generated lines are posted on the UM Head and Neck SPORE Tissue Core website (http://www2.med.umich.edu/cancer/hnspore/cores-tissue.cfm). The UM-SCC84 cell line has been utilized in prior studies [34], [62].

HaCaT, UM-SCC84, MDA-MB468, MDA-MB231, MCF7, 4T1, CT26, HEK293 and BT474 cells were maintained with Dulbecco modified Eagle medium with 10% fetal calf serum and 1% penicillin/streptomycin. T47D, HCC1954 and HCC1143 cells were maintained with RPMI medium with 10% fetal calf serum and 1% penicillin/streptomycin. To create stable cell lines, retrovirus infected or plasmid transfected cells were placed under antibiotic selection and individual colonies ring cloned. At least 20 individual clones were tested. Positive clones were kept under antibiotic selection (zeocin 500ug/ml). Controls were transfected/infected with a non-silencing shRNA construct (pSMP, Open Biosystems) and pooled populations kept under antibiotic selection and tested. Cells expressing non-targeting shRNA's were similar to untransfected control cells.

### Immunoprecipitation and Western blot

Cells were lysed in lysis buffer (50mM Tris HCl pH 7.5, 150mM NaCl, 5mM EDTA, 2mN Na_3_VO_4_, 100mM NaF, 10mM NaPPi, 10% glycerol, 1% Triton X-100), membranes pelleted by centrifugation and soluble proteins assayed by BCA protein assay (Pierce, Rockford, IL). Equal total protein was used for IP and IB analysis.

### Antibodies

IP Antibodies include: Santa Cruz anti-EphrinB1 (sc-1011, Santa Cruz, CA), Dako anti-ErbB2 (Carpinteria, CA), Sigma anti-EphrinB1 (E5404). IB antibodies used: Sigma anti-HA clone HA-7), Sigma anti-FLAG, Dako anti-ErbB2, Invitrogen anti-ErbB2(Carlsbad, CA), Sigma anti-EphrinB1, Santa Cruz anti-EphrinB1, Cell Signaling anti-Phospho-Erk1/2 (Danvers, MA), Calbiochem anti-Erk1/2 (San Diego, CA), Ambion anti-GAPDH (Austin, TX), Millipore anti-Phospho-tyrosine (4G10, Billerica, MA), Santa Cruz anti-PTPN13 (H-300), Cell Signaling anti-phospho-Src (Tyr416). The anti-ErbB2 antibodies detect wildtype ErbB2 and mNeuNT. Antibodies for immunofluorescence were: Invitrogen anti-ErbB2, Cell Signaling anti-phospho-EphrinB, Sigma anti-HA, Sigma anti-FLAG, Dako anti-ErbB2 and Santa Cruz anti-EphrinB1. Surface EphrinB ligands were bound by EphB1-Fc (R&D Systems, Minneapolis, MN) and detected with Millipore anti-human IgG-FITC.

For IP, soluble proteins were incubated with antibody, complexed to protein G agarose beads (ThermoScientific, Rockford, IL), washed with lysis buffer and pelleted by centrifugation. Complexes were dissociated with sample buffer (4% SDS, 100mM DTT, 20% glycerol, 0.005% bromophenol blue, 0.065M Tris pH 6.8), separated by SDS-PAGE , transferred to PVDF membranes (Immobilon-P, Millipore), blocked with either 5% Bovine Albumin Fraction V (Millipore) or 5% milk (Carnation instant non-fat dry milk), washed in TTBS (0.05% Tween-20, 1.37M NaCl, 27mM KCl, 25mM Tris Base), and incubated in primary antibody. Washed membranes were incubated with HRP-conjugated secondary antibody, incubated with chemiluminescent substrate (ThermoScientific, SuperSignal West Pico) and exposed to film (GeneMate Blue Light Autorad Film).

### Immunocytochemistry

Cells were seeded on collagen-coated 8 well chamber-slides, fixed with 4% paraformaldehyde (Electron Microscopy Sciences, Hatfield, PA), permeabilized with 0.2% TritonX-100 (Thermo Scientific), blocked with Superblock (Pierce) and incubated with antibody (1∶100). Following phosphate buffered saline (PBS) washes, cells were incubated with Alexa Fluor-conjugated secondary antibody (Invitrogen), washed, coverslips mounted with Vectashield mounting medium plus DaPi (Vector Labs, Burlingame, CA) and cells analyzed by confocal microscopy (Olympus FluoView1000). For surface EphinB localization, cells on chamber-slides were placed on ice, EphB-Fc bound to the surface for 2 hours. Following PBS washes, cells were fixed and bound EphB-Fc detected with IgG-FITC.

### Src inhibition

PP2, an inhibitor of the Src kinase family, blocks Src function in the 1–25 µM range [36], [63]. Cells were treated with 10 µM PP2 (Calbiochem) for 4 hours at 37°C and cell lysates were harvested as described. Control cells were treated with vehicle (DMSO).

Saracatinib (AZD-0530, LC Laboratories, Woburn, MA) is a dual specific Src/Abl kinase inhibitor. Cells were treated with either vehicle alone (DMSO), 0.25, 0.5 or 1.0 µM saracatinib for 3 hours at 37°C and cell lysates harvested and analyzed by IP and western blot.

### Breast Tumor Array

Gene expression array data from early stage breast cancers measured on Affymetrix U133A (published by Wang *et al* [64]) and Affymetrix U133 2 Plus chips (published by Richardson *et a*l [65]) were combined and analyzed. The dataset of Richardson *et al* was made compatible with that of Wang *et al* by restricting it to the probe sets of the U133A chip and processing it with the mas5 software available at http://www.bioconductor.org. Systematic source and batch bias adjustment in the two datasets was performed by the distance-weighted discrimination (DWD) method, suitable for the correction of systematic biases associated with micro array data sets [66]. Robust consensus clustering techniques were used to classify the breast cancer cases into basal-like cancers (BL), HER2+, Luminal A, Luminal B and normal breast [67]. The average expression of each gene across all samples was normalized to 0. The mean relative expression of probes corresponding to gene of interest in each subtype was calculated and graphed. Statistical significance was analyzed by two-tailed T-test.
